# Analysis of S gene characteristic sequences and changes in properties of protein expression in HBV ASCs with low-level HBsAg

**DOI:** 10.3389/fmed.2022.948842

**Published:** 2022-09-14

**Authors:** Yu Yu, Yingqiang Zhang, Yuzhu Dai, Qingyang Sun, Chun Jiang, Xujian Xu, Chuanzhong Mei, Jun Cheng

**Affiliations:** ^1^School of Laboratory Medicine, Bengbu Medical College, Bengbu, China; ^2^Department of Clinical Research, The 903rd Hospital of PLA, Hangzhou, China; ^3^Department of Clinical Laboratory, The Affiliated Suzhou Hospital of Nanjing Medical University, Suzhou Municipal Hospital, Gusu School, Nanjing Medical University, Suzhou, China; ^4^Department of Biotechnology, The University of Tokyo, Tokyo, Japan; ^5^Faculty of Graduate Studies, Jiangsu University, Zhenjiang, China

**Keywords:** HBsAg, HBV S gene, HBV genotype, MHR, mutation site, HBsAg function

## Abstract

**Objective:**

We detected the serum HBsAg immune complex (HBsAg-CIC) and sequenced the HBV S gene in these patients to reveal the association between sustained low-level expression of HBsAg and mutated S gene sequence characteristics, protein function changes, and HBsAg immune complex formation.

**Methods:**

A total of 204 samples were collected and divided into high-level (*n* = 60, HBsAg level >10 IU/ml) and low-level (*n* = 144, HBsAg level ≤ 10 IU/ml) HBsAg groups. The clinical and epidemiological data of the two groups were statistically compared. According to different serological patterns and genotypes, the HBsAg-CIC results of the high-level and low-level HBsAg groups were divided into different subgroups, and then the HBsAg-CIC positive rates among different subgroups were compared. We sequenced the S gene of HBV from the two groups and identified the relevant mutations in the MHR of the S gene. In addition, we compared the changes in HBsAg protein properties and functions after hot spot mutation in the MHR of the S gene.

**Results:**

Comparing the positive rates of HBsAg-CIC under different serological patterns and genotypes in the two groups, the HBsAg-CIC positive rate was higher in the low-level HBsAg group. Moreover, there was weak correlation between HBsAg-CIC and HBsAg or HBV DNA in both groups (*r* = 0.32, 0.27, 0.41, 0.48; *P* < 0.05). Sequencing of S gene in the two groups, showed that the hot-spot mutations were T126A, M133L/T/S, and F134L/T/I in MHR of S gene of genotype B, and hot-spot mutations were Q101R and I126S/T in MHR of S gene of genotype C. Additionally, the positive rate of MHR mutation in the S gene from HBsAg-CIC positive patients was higher in the low-level HBsAg group.

**Conclusion:**

The host immune process of clearing HBV seems to have multiple site mutations in MHR, which changes the physicochemical properties and functions of HBsAg and intensifies the formation of HBsAg-CIC, thus avoiding the effective recognition of HBsAg by the host and resulting in immune tolerance between the host and HBV, which may be one of the formation mechanisms of sustained low-level expression of HBsAg in the serum of HBV-infected persons.

## Introduction

Hepatitis B virus (HBV) infection is one of the most serious problems endangering human health. At present, approximately 240 million people in the world are infected with HBV (1, 2). HBV is mainly prevalent in Asia, the Pacific Islands, Africa, southern Europe and Latin America, and there are more than 100 million HBV carriers in China (3–5). Based on the interaction between the virus and host immunity, HBV infection presents a variety of clinical manifestations, including acute hepatitis, chronic hepatitis, liver dysfunction, liver cirrhosis and hepatocellular carcinoma (6). Some asymptomatic HBV-infected people have persistent low-level expression of HBsAg, and the literature reports show that the proportion is as high as 15.03–21.1% (7, 8). However, some patients with low-levels expression of HBsAg often miss detection in the process of clinical detection, which undoubtedly does not increase the risk of HBV transmission and poses a serious threat to the safety of clinical blood transfusion.

HBsAg is typically used in the clinical diagnosis and screening of HBV-infected persons as an important serological marker. In addition, it was reported that the level of HBsAg was closely related to the disease stage, disease progression and prognosis of HBV-infected patients (9, 10). The level of HBsAg in the serum of HBV-infected patients depends not only on the process of virus replication but also on the expression of the corresponding coding mRNA and the complex balance of the interaction between HBV and the host immune system (8, 11, 12). HBV-infected people with persistent low-level expression of HBsAg often have low replication of HBV DNA, and most of these people are asymptomatic HBV-infected people (13–19). Unlike occult hepatitis infection, the mechanism of chronic HBV infection with persistent low-level HBsAg expression and occult hepatitis infection partially intersect, but the low-level HBsAg population can detect the low concentration of HBsAg in the host serum, accompanied by low or no replication of HBV DNA, while the occult hepatitis infected people show negative HBsAg detection and positive HBV DNA (20). So far, the mechanism of sustained low-level expression of HBsAg in HBV-infected patients has not been fully clarified, but it is closely related to host and virus factors and virus gene mutation is an important factor (8, 12, 21, 22). HBsAg is composed of 226 amino acid (aa) residues encoded by the HBV S gene. The region from aa 99 to aa 169 is called the major hydrophilic region (MHR) and is an important antigen epitope to stimulate B cells to produce neutralizing antibodies (23, 24). Mutation of the HBV S gene, especially the MHR region, can cause immune escape by changing the antigenicity of HBsAg and reducing the binding force of neutralizing antibodies or affecting the secretion of HBsAg (25, 26). The existence of HBV-infected people with persistent low-level expression of HBsAg poses a new challenge to the prevention and treatment of HBV and has attracted extensive attention from experts in the field of infectious diseases (27–30). At present, there are few reports on the distribution of HBsAg-CIC in the serum of HBV-infected persons with sustained low-level expression of HBsAg. Moreover, we investigated whether the S gene mutation causes a change in HBsAg protein properties and whether the change in HBsAg protein properties will increase the formation of HBsAg-CIC in the serum of HBV-infected persons with sustained low-level expression of HBsAg. With these problems, we carried out the following research.

## Materials and methods

### Study design and samples

A randomized controlled study was conducted from February 2014 to December 2015 at the Clinical Laboratory Center at the 903rd Hospital of the PLA (Hangzhou, China). In total 244 serum samples of chronic asymptomatic HBV carriers (ASCs) from the 903rd Hospital of the PLA, the First Affiliated Hospital of the Medical College of Zhejiang University and Hangzhou Xixi Hospital were collected. The patients were all from the physical examination center of the hospital, and were found during the normal physical examination. Definition of chronic ASC is characterized by the presence of positive serum HBsAg for more than 6 months, normal serum amino transferase levels and no evidence of liver cirrhosis (LC) or hepatocellular carcinoma (HCC) based on the clinical criteria and ultrasound examination (31). The study was carried out in accordance with the principles of the Declaration of Helsinki. All patients signed informed consent, and the project was approved by the medical ethics committee of the 903rd Hospital of the PLA service support force.

Based on laboratory test results, as well as more than 1 year of follow-up of the patients, their clinical data and their history of infection or natural history the exclusion criteria were established (32, 33).

A total of 204 samples (40 cases were excluded) were collected and divided into high-level (*n* = 60, HBsAg level >10 IU/ml) and low-level (*n* = 144, HBsAg level ≤ 10 IU/ml) HBsAg groups. The clinical testing center of the National Health Commission of China has provided a serum standard with a low-level fixed value, the CMIA method replaced the traditional ELISA method for quantitative detection of serum HBsAg, low-level HBsAg was defined as serum HBsAg <5.0 ng/ml or ≤ 10.0 IU/ml (34). According to HBV serum markers, HBV-infected patients were divided into three serological patterns: HBsAg/HBeAg/anti-HBc positive, HBsAg/anti-HBe/anti-HBc positive, and HBsAg/anti-HBc positive. Blood samples (5 ml) were collected with a sampling tube without coagulant, and 3 ml serum samples were centrifuged at 3,000 rpm. One milliliter of serum sample was placed in three 1.5 ml EP tubes and frozen at −70°C until use.

### Experimental reagents and instruments

The following clinical and laboratory data of the included patients were recorded: age, sex, Hepatitis B surface antigen (HBsAg), Hepatitis B surface antibody (anti-HBs), Hepatitis B e antigen (HBeAg), Hepatitis B e antibody (anti-HBe), Hepatitis B core antibody (anti-HBc), HBV DNA, among other markers. Architect i2000 automatic chemiluminescence immunoanalyzer based on chemiluminescence immunoassay (CLIA) (Abbott Laboratories, USA) were used. HBV DNA fluorescence quantitative detection kit was purchased from ACON Biotechnology Co., Ltd (Hangzhou, China). HBV DNA was detected using ABI 7300plus real-time fluorescent PCR system (ABI Applied System, USA) and Np968 nucleic acid extraction system (Tianlong, China).

### Circulating immune complex (CIC) detection

In this study, HBsAg-CIC was detected (HBsAg-CIC related detection results in this study were completed when the samples were collected) based on the self-developed patented immune complex dissociation Technology (which has the advantages of high efficiency, versatility, strong specificity and high sensitivity; protocol no. ZL201410034039.5 and ZL201410033277.4). The CIC detection method used in this study consists of the following steps: separation, washing, redissolution, detection, result judgment. HBsAg-CIC dissociation was performed in the serum of HBV-infected patients according to the literature and patented technology, and presence of HBsAg-CIC was judged according to the result judgement standard (35). The samples of the high-level and low-level HBsAg groups were grouped according to different serological models and genotypes. The positive rates of HBsAg-CIC were compared, and the correlation between the content of dissociated HBsAg in HBsAg-CIC and the content of free HBsAg in serum was evaluated.

### Comparison and analysis of HBV gene mutations

Gene sequencing was performed on serum samples from 144 asymptomatic infected persons in the low-level group and 60 asymptomatic infected persons in the high-level group. Nested PCR was used to amplify HBV nucleic acids in high- and low-level HBsAg groups, and product recovery sequencing was performed (8, 12). Seqman, a subroutine of Laser gene software, was used to splice the sequencing results, and MEGA software was used to compare the spliced S genes with S genes of different genotypes to identify genotypes with different sequencing results (36). Based on the reference sequence of the S gene reported in the literature, the frequency of mutation at different sites of the S gene in the high and low HBsAg groups was counted, and the frequency of mutation at the same location between the two groups was compared and verified. In addition, the mutation of the HBV S gene in HBsAg-CIC samples between the two groups was statistically analyzed. A mutation rate >10% is defined as a hot spot mutation (8, 12, 37).

### Functional analysis of HBsAg

Protean of Lasergene software (DNAstar, Inc., Madison, WI, USA) was applied to predict the effect of amino acid mutations in the MHR region in individuals in the low-level HBsAg group with genotype B on the following parameters: the HBsAg hydrophilicity using a Kyte-Doolittle hydropathy plot; the HBsAg antigenic index using the Karplus-Schultz method; and the HBsAg surface probability using the Jameson-Wolf and Emini method. Functional changes in HBV MHR (“a” antigenic determinant as a major detection site) of individuals in the low HBsAg group with genotype B were analyzed based on the reference sequences from individuals in the high-level HBsAg group with genotype B.

### Statistical analysis

For statistical analysis of the data used, SPSS 19 software was used, and the data were imported into Graph Pad Prism5 software to complete the drawing of the bar graph. Canvas 11 image editing software was used to draw a Venn diagram. The continuous variables and categorical variables between the two groups were statistically analyzed by *t-*test, chi square test or Fisher exact test. Correlation analysis was performed using Pearson Product-Moment Correlation. All *P*-values were double tailed. When *P* < 0.05, the difference between two or more groups was considered statistically significant.

## Results

### Comparison of routine laboratory data between the two groups

In Table 1, the results showed that there were significant differences in HBV genotype (B genotype was the main genotype in the low-level HBsAg group), HBV DNA positive rate (was lower in the low-level HBsAg group) and age (was higher in the low-level HBsAg group) between the two groups (*P* < 0.05), but there was no significant difference in sex between the two groups (*P* > 0.05).

**Table 1:** Clinical data and virological characteristics of the low- and high-level HBsAg chronicASC groups.

**Parameter**	**Low-level HBsAg group (*n* = 144)**	**High-level HBsAg group (*n* = 60)**	***P***
**Gender**			
Male	92	39	>0.05
Female	52	21	
Age (years)	54.88 ± 14.93	42.67 ± 12.19	<0.05
**Laboratory results**			
HBsAg (IU/ml)	4.08 ± 3.56	5406.49 ± 12885.12	<0.05
HBV DNA-positive rate (%)	47.92 (69/144)	95.00 (57/60)	<0.05
**HBV genotype**			
B genotype (%)	36.11 (52/144)	40.00 (24/60)	<0.05
C genotype (%)	7.64 (11/144)	38.33 (23/60)	

### Distribution of HBsAg-CIC among asymptomatic HBV infections

Comparing the HBsAg-CIC positive rates in different genotypes and serological patterns of the two groups (Table 2), the results showed that the HBsAg-CIC positive rate between the two groups was statistically significant (*P* < 0.05), and in the low-level HBsAg group, the HBsAg-CIC positive rate was higher than in the high-level HBsAg group. Under the condition of the same B genotype, the HBsAg-CIC positive rates between the two groups were statistically significant (the HBsAg-CIC positive rate was higher in the low HBsAg group, *P* < 0.05). In addition, the difference in the HBsAg-CIC positive rate in different serological patterns was statistically significant in the high-level HBsAg group (*P* < 0.05), and the HBsAg-CIC positive rate was the highest among the serological patterns of HBsAg/HBeAg/anti-HBC positivity. In the high-level HBsAg group, the difference in the HBsAg-CIC positive rate in different genotypes was statistically significant (*P* < 0.05), and in the C genotype, the HBsAg-CIC positive rate was higher.

**Table 2:** Results of HBsAg-CIC positive rate between different serological patterns and genotypes in the two groups.

**Parameter**	**Low-level HBsAg group (*n* = 144)**	**High-level HBsAg group (*n* = 60)**	***P***
HBsAg/HBeAg/anti-HBc positive	/	15 (21)^a^	/
HBsAg/anti-HBe/anti-HBc positive	105 (144)	7 (21)^a^	<0.05
HBsAg/anti-HBc positive	/	9 (21)^a^	/
Total	105 (144)	31 (60)	<0.05
Genotype B	32 (52^b^)^d^	8 (24^c^)^e^	<0.05
Genotype C	10 (11^b^)^d^	18 (23^c^)^e^	>0.05

“/”: No relevant data; “a”: In the high-level group, the difference in the HBsAg-CIC positive rate between different HBV serological modes was statistically significant (χ^2^=6.94, P < 0.05); “b”: The samples of 144 patients in the low-level group were successfully classified into 52 cases of B genotype and 11 cases of C genotype by HBV gene sequencing; “c”: The samples of 60 patients in the high-level group were successfully classified into 24 cases of B genotype and 23 cases of C genotype by HBV gene sequencing; “d”: There was no significant difference in the HBsAg-CIC positive rate between genotype B and genotype C in low-level group (χ^2^=3.52, P>0.05); “e”: There was significant difference in the HBsAg-CIC positive rate between genotype B and genotype C in high-level group (χ^2^=11.37, P < 0.05).

### Evaluation of the correlation between HBsAg CIC and HBsAg or HBV DNA between the two groups

**Figure 1:**
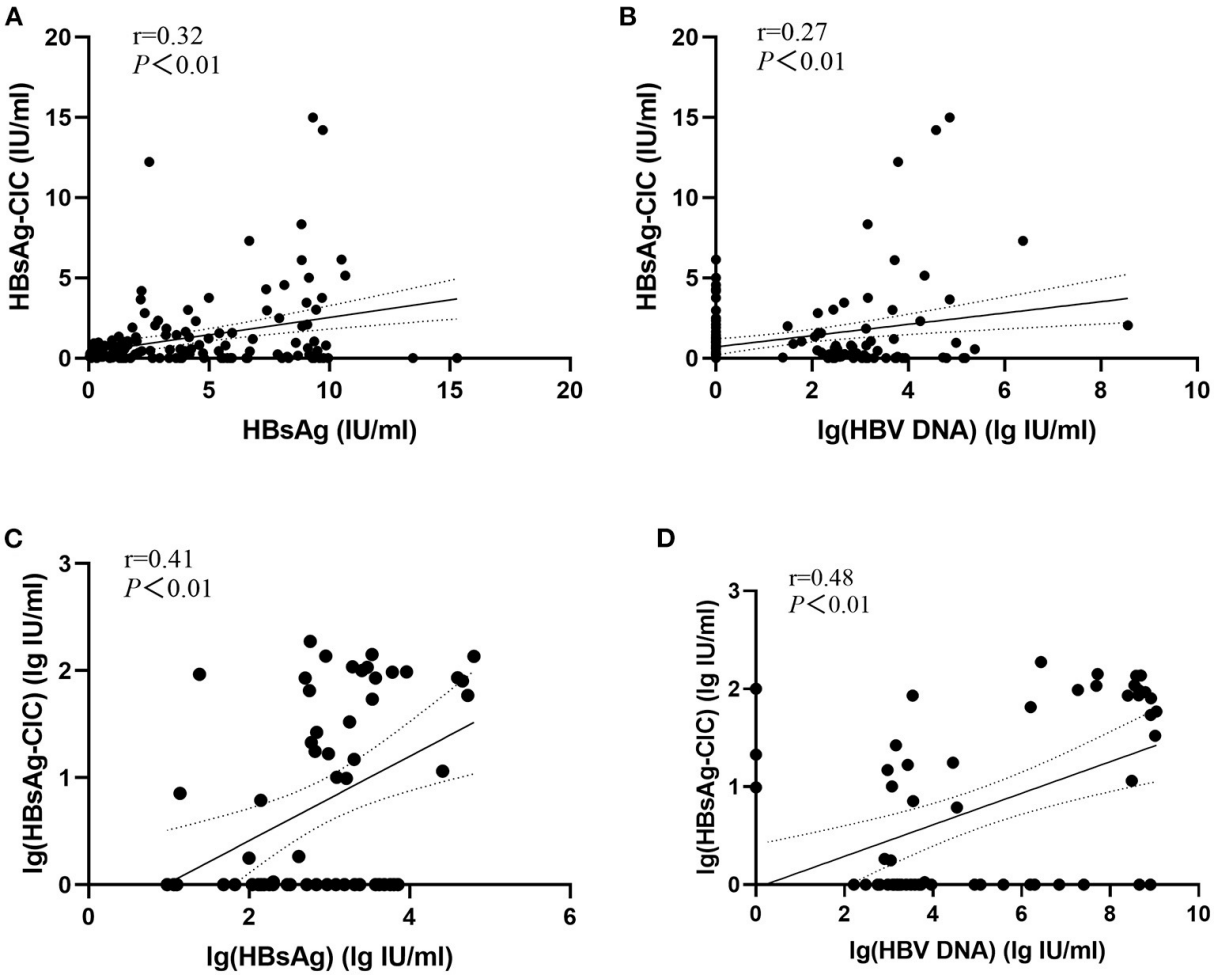
Evaluation of the correlation between HBsAg-CIC and HBsAg or HBV DNA between the two groups. **(A)** Linear regression model of HBsAg-CIC and HBsAg in patients with low-level HBsAg. **(B)** Linear regression model of HBsAg-CIC and lg(HBV DNA) in patients with low-level HBsAg. **(C)** Linear regression model of lg(HBsAg-CIC) and lg(HBsAg) in patients with high-level HBsAg. **(D)** Linear regression model of lg(HBsAg-CIC) and lg(HBV DNA) in patients with low-level HBsAg.

The correlation between HBsAg-CIC and HBsAg or HBV DNA between the two groups was evaluated as shown in Figure 1. In the low-level HBsAg group, HBsAg-CIC has a weak correlation with HBsAg and lg(HBV DNA) (*r* = 0.32, 0.27; *P* < 0.05). In the high-level HBsAg group, lg(HBsAg-CIC) had also weak correlation with lg(HBsAg) and lg(HBV DNA) (*r* = 0.41, 0.48; *P* < 0.05).

### Evaluation of the relationship between mutation in the MHR of the S gene and the HBsAg-CIC positive rate

**Figure 2:**
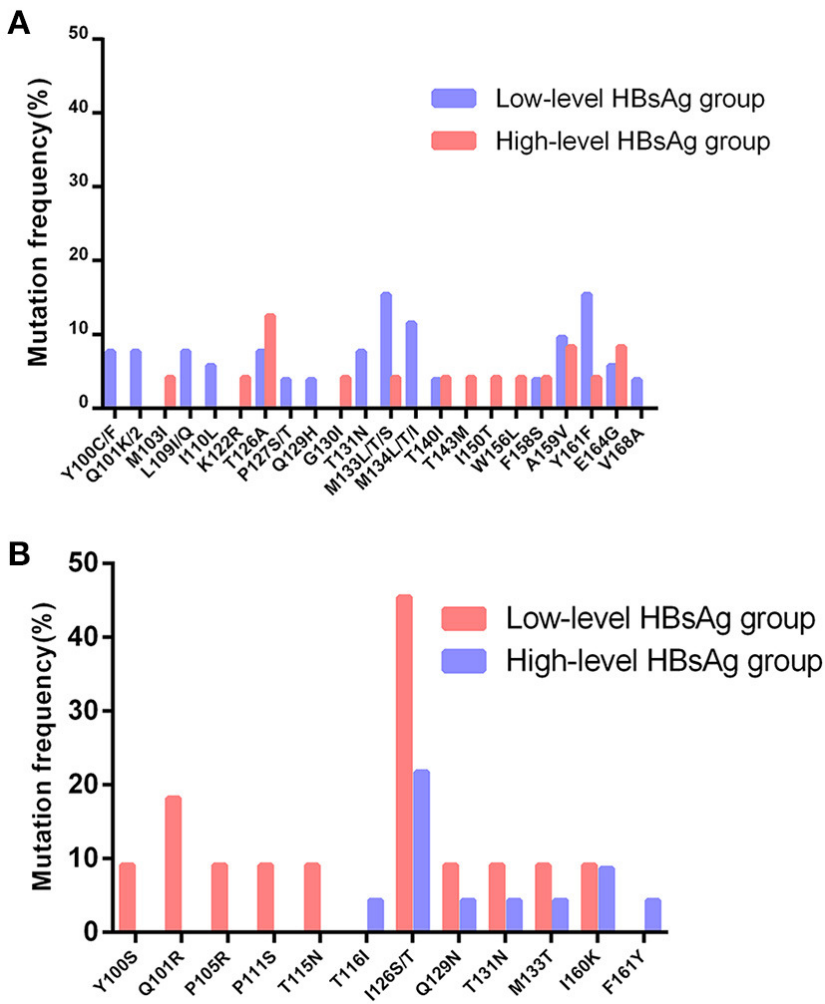
Distribution and rate of mutations within MHR in S gene of genotype B and C between the two groups. **(A)** Distribution and rate of mutations within MHR in S gene of genotype B between the two groups. **(B)** Distribution and rate of mutations within MHR in S gene of genotype C between the two groups.

The S gene sequencing results of the two groups were compared with the reference sequences of corresponding genotypes (8, 12), as shown in Figure 2. The results showed that the hot spot mutations in the S gene MHR of HBV genotype B patients were T126A, M133L/T/S, and F134L/T/I. Hotspot mutations in the MHR of the S gene of HBV genotype C in the two groups were Q101R and I126S/T.

**Figure 3:**
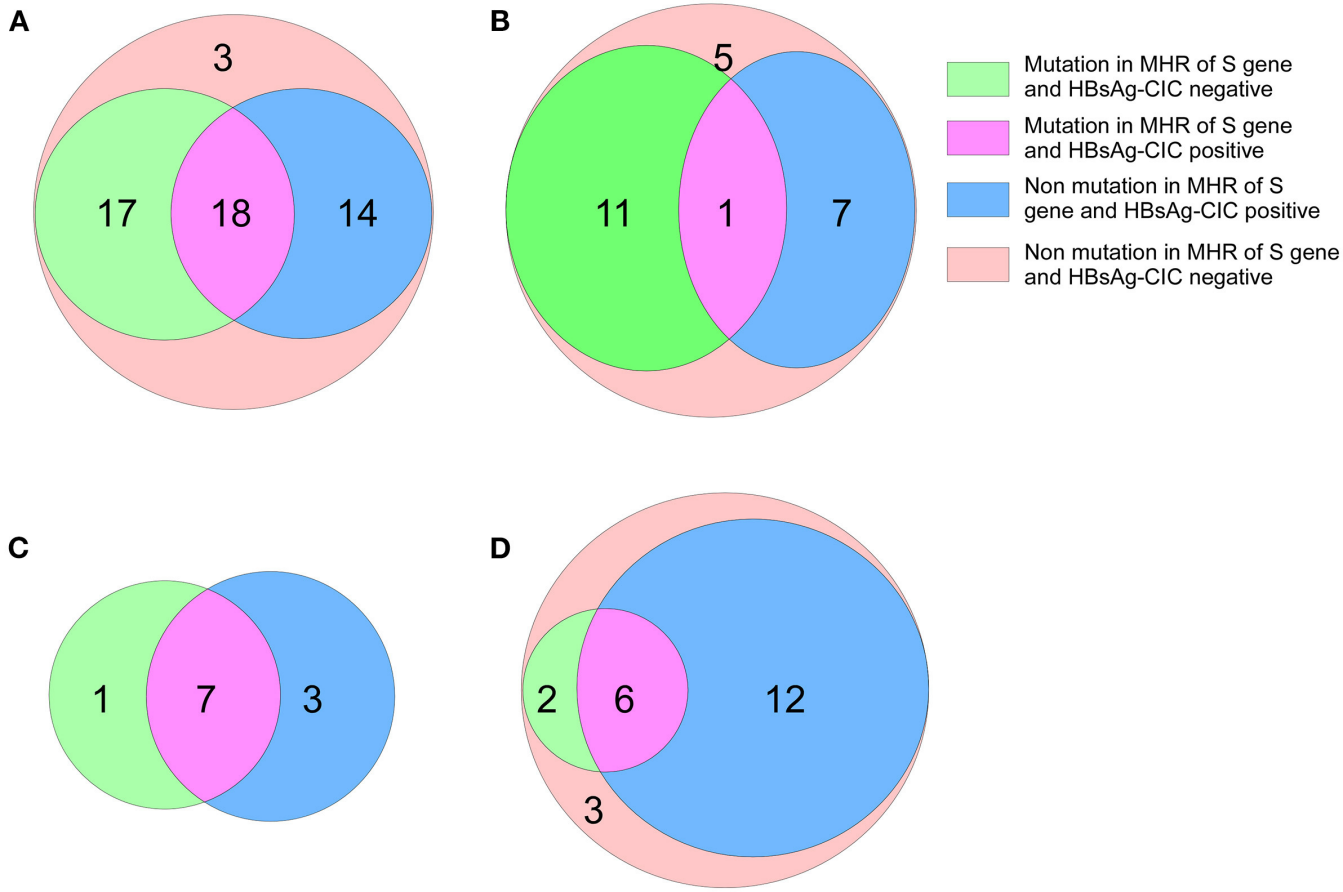
Mutation in MHR of S gene and distribution of HBsAg-CIC in patients with different genotypes between the two groups. **(A)** Mutation in MHR of S gene and distribution of HBsAg-CIC positive of genotype B in patients with low-level HBsAg. **(B)** Mutation in MHR of S gene and distribution of HBsAg-CIC positive of genotype B in patients with high-level HBsAg. **(C)** Mutation in MHR of S gene and distribution of HBsAg-CIC positive of genotype C in patients with low-level HBsAg. **(D)** Mutation in MHR of S gene and distribution of HBsAg-CIC positive of genotype C in patients with high-level HBsAg.

In addition, 32 patients with genotype B in the low-level HBsAg group were found to be HBsAg-CIC positive, and 18 had MHR mutations of the S gene. Among the patients in the high-level HBsAg group, 8 cases were HBsAg-CIC positive for genotype B, and 1 case had an MHR mutation of the S gene. The positive rate of MHR mutation in the S gene in HBsAg-CIC-positive patients with the B genotype was significantly different between the two groups (χ^2^ = 4.91, *P* < 0.05). Ten cases were HBsAg-CIC positive for genotype C in the low-level HBsAg group, and 7 cases had MHR mutations. In the low-level HBsAg group, 18 cases were HBsAg-CIC positive for genotype C, and 6 cases had MHR mutations (Figure 3).

### Functional analysis of HBsAg

**Figure 4:**
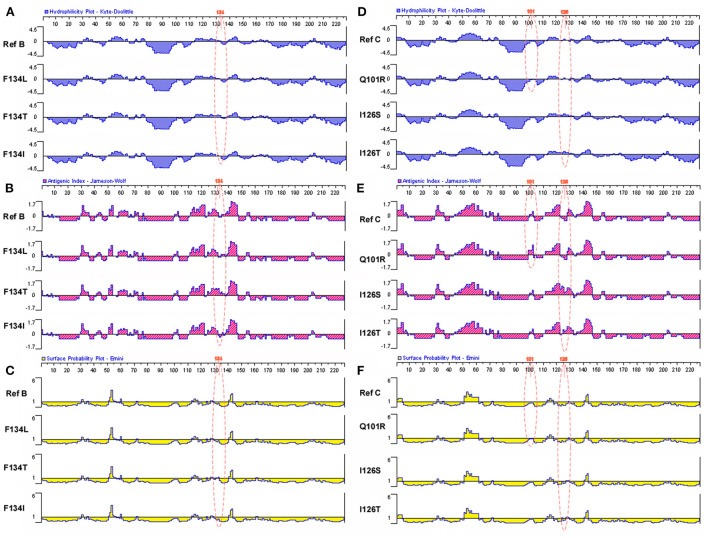
Related functional changes induced by amino acid mutations in MHR region. **(A)** Effects of hotspot mutations (T126A, M133L/T/S, and F134L/T/I) in the genotype B with low-level HBsAg group on HBsAg antigen index. **(B)** Effects of hotspot mutations (T126A, M133L/T/S, and F134L/T/I) in the genotype B with low-level HBsAg group on HBsAg hydrophilicity. **(C)** Effects of hotspot mutations (T126A, M133L/T/S, and F134L/T/I) in the genotype B with low-level HBsAg group on HBsAg surface probability. **(D)** Effects of hotspot mutations (Q101R and I126S/T) in the genotype B with low-level HBsAg group on HBsAg antigen index. **(E)** Effects of hotspot mutations (Q101R and I126S/T) in the genotype B with low-level HBsAg group on HBsAg hydrophilicity. **(F)** Effects of hotspot mutations (Q101R and I126S/T) in the genotype B with low-level HBsAg group on HBsAg surface probability.

In this study, the hydrophilicity, antigenic index, and surface probability of mutational sites and surrounding amino acid sites in the MHR region of individuals with HBV genotype B in the low-level HBsAg group were analyzed to predict the functions of these MHR sites (Genotype B: T126A, M133L/T/S, F134L/T/I, Genotype C: Q101R, I126S/T). Compared with the reference sequence, we found that in the low-level HBsAg group, the changes in multiple sites and multiple areas were related to hydrophilicity, the antigenic index and surface probability. It will not only affect the binding between HBsAg and the detection antibody, resulting in the deviation of the detection results, but also affect the host's effective recognition of HBsAg, resulting in immune escape and continuous immune tolerance (Figure 4).

## Discussion

At present, the preferred serological marker for the diagnosis of HBV infection is hepatitis B surface antigen (HBsAg) (38, 39). It is the earliest serological marker after HBV infection and can be detected 4–10 weeks after HBV infection. HBsAg mainly consists of large (L), medium (M) and small (S) surface antigens. The smallest surface antigen, S protein (24 kDa), is 226 amino acids in length. HBsAg is very important to monitor the natural process, evaluate the treatment response and predict stages of disease progression (40, 41). Previous studies have shown that some patients with HBV infection continue to express HBsAg at low levels, and this population has unique clinical manifestations and epidemiological characteristics. In this study, we grouped and compared the results of routine laboratory examinations of patients. It was found that HBV-infected patients with persistent low-level expression of HBsAg had the characteristics of older age, B genotype, HBsAg/anti-HBe/anti-HBC positivity, and HBV DNA low replication, which was consistent with previously reported studies (8, 12).

Based on immune complex dissociation technology independently established by our project team (the technology has the advantages of high efficiency, generality, strong specificity, and high sensitivity), HBsAg-CIC of high- and low-level HBsAg groups was detected in this study. The results showed that HBsAg/HBeAg/anti-HBC positive had a higher HBsAg-CIC positive rate than the other two serological patterns, which was consistent with Tsai et al. (42). The reason is that there is a positive correlation between the HBsAg-CIC positive rate and HBeAg. In other words, it is closely related to virus replication. In different genotypes of the high-level HBsAg group, the difference in the HBsAg-CIC positive rate was statistically significant, and the HBsAg-CIC positive rate of genotype C was higher than that of genotype B. In different genotypes of the low-level HBsAg group, the difference in the HBsAg-CIC positive rate was not statistically significant. This may be related to the fact that in the low-level HBsAg group, the number of samples of the C genotype was low. After reviewing many studies, no investigation report on different HBV genotypes of HBsAg-CIC has been found, but the literature clearly indicates that the proportion of HBV genotype C is significantly higher than that of HBV genotype B in HBeAg-positive patients. Consistent with this study, 70% (14/20) of HBV infections with serological pattern HBsAg/HBeAg/anti-HBc positive were C genotype. This indirectly indicates that investigating the HBsAg-CIC positive rate among HBV patients with different genotypes is reliable.

In addition, this study also evaluated the correlation between HBsAg-CIC and HBsAg or HBV DNA between the two groups. The results showed that in the low-level group and high-level group, the content of HBsAg-CIC was weak positively correlated with the content of serum HBsAg and HBV DNA, suggesting that the formation of HBsAg-CIC was closely related to virus replication and the effective recognition of antigen epitopes of serum HBsAg. However, this study also found that when the serological pattern was the same, the positive rate of HBsAg-CIC in the low-level HBsAg group was higher than that in the high-level HBsAg group. HBV infected persons with HBeAg positive and genotype C have active viral replication and high content of free HBsAg, which in turn stimulates the body to produce HBsAg-CIC in a higher level than other serological patterns and B genotypes. The elimination of HBV referred to in this discussion actually referred to the elimination of HBsAg-CIC. In other words, compared with the high-level HBsAg group, why is the HBsAg-CIC positive rate higher in the low-level HBsAg group than in the high-level HBsAg group under the condition of low replication. We think that in the high-level group, especially those with HBeAg positive and genotype C HBV infection, the body may play the role of HBsAg-CIC clearance normally, but its clearance capacity is insufficient compared with the amount of HBsAg-CIC formation. However, although the formation of HBsAg-CIC was low in the low-level HBsAg group, the body did not exert the function of clearing HBsAg CIC or the ability of effectively clearing HBsAg CIC decreased which led to the accumulation of HBsAg-CIC, resulting in a higher positive rate of HBsAg-CIC in the low-level group (43). This conclusion needs further experimental verification.

Relevant studies show (13, 17) that there is unexplained immune tolerance between HBV and the host, or the application of immunosuppressants enhances the immune tolerance of the body, so that HBsAg cannot be completely cleared, resulting in the expression of low-level HBsAg. In addition, S gene methylation, pre-core region (PC) and basal core promoter (BCP) mutations regulate the secretion of HBsAg, resulting in lower HBsAg levels in patients. To this end, we carried out further research, obtained the distribution of hotspot mutations in MHR by sequencing the S genes of the two groups, and then counted the number of HBsAg-CIC-positive patients with S gene MHR mutations in different genotypes in the two groups. After statistical comparison, we found that in the low-level HBsAg group, the frequency of MHR in-mutation in HBsAg-CIC-positive patients of the B genotype was higher than that in the high-level HBsAg group, while the difference in the frequency of MHR in-mutation in HBsAg-CIC-positive patients of the C genotype between the two groups was not significant. This may be related to fewer HBV infection cases of genotype C. In addition, based on the immune interaction between the virus and the host (immune surveillance and immune escape), we believe that the gene mutation in the low-level HBsAg group is higher than that in the high-level group in the S gene, may be that the formation of the low-level HBsAg is more conducive to the long-term coexistence between the virus and the host, which needs to be further demonstrated (7, 44).

From the above results, we can indirectly conclude that the HBsAg-CIC positive rate in the low-level HBsAg group was higher, which may be related to the mutation in the MHR of the HBV S gene. We evaluated the antigenicity, hydrophilicity and other related parameters of HBV genotypes B and C with hot spot mutations in the MHR of the S gene. The study found that mutations occurring within the MHR hotspot may change the antigenicity and hydrophilicity of HBsAg in the corresponding position, which increases the antigenicity and hydrophobicity of the HBsAg part locus mutation. There were also mutations that reduced antigenicity and hydrophobicity at some HBsAg sites. Combined with our previous studies, the low expression level of HBsAg in HBV-infected persons may be a dynamic balance between the host and HBV, thus limiting the further effective elimination of HBV by the host. In addition, it has been reported that mutation of the S gene in MHR may be related to HBV immune escape (45). We speculated that the formation of HBsAg-CIC may be the process of active elimination of HBV by the host, but the virus escaped from the MHR mutation and was further cleared by the host. Multiple factors lead to a higher proportion of HBsAg-CIC in HBV-infected patients with low levels of HBsAg, which further promotes the continuous low level of HBsAg expression. Therefore, whether the HBsAg-CIC positive rate in the low-level HBsAg group was higher than that in the high-level HBsAg group may be related to the change in HBsAg protein properties caused by mutations in MHR. Whether the formation of HBsAg-CIC is an influential factor for HBV-infected patients with continuously low levels of HBsAg expression needs to be verified by subsequent experiments.

Although a large number of samples were collected and grouped in this study, we speculated that the sustained low-level expression of HBsAg may be related to the mutation of multiple sites in the MHR region of S gene and the formation of HBsAg-CIC, but this study still has some limitations. For example, (1) there are few samples of HBsAg-CIC positive patients with different genotypes in patients with continuous low-level expression of HBsAg, which cannot fully confirm the correlation between multi-site mutation in MHR region of S gene and the formation of HBsAg-CIC in patients with different genotypes of HBV, which needs to be verified by further experiments. (2) Part of this study is based on bioinformatics, which cannot be fully and truly reflect the changes of HBsAg related characteristics caused by S gene mutation, which needs to be verified by further experiments.

In conclusion, a large-scale grouping study was conducted on 204 patients with ASC. The results of these analyses show that the potential mechanism of sustained low levels of HBsAg expression, which is the process of host clearance of HBV immunity, seems to involve multiple site mutations in MHR, which changes the physical and chemical properties and functions of HBsAg, intensifies the formation of HBsAg-CIC, avoids the effective recognition of HBsAg, and leads to immune tolerance between the host and HBV.

## Data availability statement

The raw data supporting the conclusions of this article will be made available by the authors, without undue reservation.

## Ethics statement

The studies involving human participants were reviewed and approved by the Medical Ethics Committee of the Hospital (the 903rd Hospital of the PLA) (Protocol No: PLA-117-20160309). The patients/participants provided their written informed consent to participate in this study.

## Author contributions

CM participated in the project design and research, performed the statistical analysis, and was responsible for drafting and revision of the manuscript. YD participated in the project design and coordination, assisted in writing the manuscript, and helped with the statistical analysis. YQZ was responsible for sample collection. YY and JC performed the virologic analysis and helped draft the manuscript. XX and CJ performed the molecular genetic analysis and sample collection. YY performed the virologic analysis and sample collection and participated in the statistical analysis. All authors read and approved the final manuscript.

## Conflict of interest

The authors declare that the research was conducted in the absence of any commercial or financial relationships that could be construed as a potential conflict of interest.

## Publisher's note

All claims expressed in this article are solely those of the authors and do not necessarily represent those of their affiliated organizations, or those of the publisher, the editors and the reviewers. Any product that may be evaluated in this article, or claim that may be made by its manufacturer, is not guaranteed or endorsed by the publisher.

## References

[1] Wang S Sun Y Wang Y Wang A Kou B Che Y. ASPP2 inhibits hepatitis B virus replication by preventing nucleus translocation of HSF1 and attenuating the transactivation of ATG7. J Cell Mol Med. (2021) 25:6899–908. 10.1111/jcmm.16699 34085409 PMC8278078

[2] Tressler S Bhandari R. Interventions to increase completion of hepatitis B vaccination in people who inject drugs: a systematic review and meta-analysis. Open Forum Infect Dis. (2019) 6:ofz521. 10.1093/ofid/ofz521 31890724 PMC6929254

[3] Ma L Zheng X Yang Y Wang J Xu Y Wang B. Epigenetic differences of chronic hepatitis B in different TCM syndromes: protocol for a case-control, non-interventional, observational clinical study. Medicine. (2018) 97:e12452. 10.1097/MD.0000000000012452 30278525 PMC6181568

[4] Wang W Dong R Guo Y He J Shao C Yi P. CircMTO1 inhibits liver fibrosis *via* regulation of miR-17-5p and Smad7. J Cell Mol Med. (2019) 23:5486–96. 10.1111/jcmm.14432 31148365 PMC6653252

[5] Zhao Z Tang H Feng L. Measles-associated severe pneumonia in a patient with HBeAg-negative chronic hepatitis B: a case report. Zoonoses. (2022) 2:4. 10.15212/ZOONOSES-2021-0013

[6] Mason WS Jilbert AR Litwin S. Hepatitis B virus DNA integration and clonal expansion of hepatocytes in the chronically infected liver. Viruses. (2021) 13:210. 10.3390/v13020210 33573130 PMC7911963

[7] Cheng J Dai Y Yan L Zhou H Xu X Sun C. Clinical characteristics and correlation analysis of subjects with chronic hepatitis B virus (HBV) infection and sustained low levels of hepatitis B surface antigen (HBsAg). Med Sci Monit. (2018) 24:1826–35. 10.12659/MSM.905445 29593208 PMC5890521

[8] Wang T Cui D Chen S Xu X Sun C Dai Y. Analysis of clinical characteristics and S gene sequences in chronic asymptomatic HBV carriers with low-level HBsAg. Clin Res Hepatol Gastroenterol. (2019) 43:179–89. 10.1016/j.clinre.2018.08.015 30293895

[9] Kuhns MC Holzmayer V McNamara AL Sickinger E Schultess J Cloherty GA. Improved detection of early acute, late acute, and occult Hepatitis B infections by an increased sensitivity HBsAg assay. J Clin Virol. (2019) 118:41–5. 10.1016/j.jcv.2019.08.001 31442662

[10] Li Y Cai Q Xie Q Zhang Y Meng X Zhang Z. Different mechanisms may exist for HBsAg synthesis and secretion during various phases of chronic hepatitis B virus infection. Med Sci Monit. (2017) 23:1385–93. 10.12659/MSM.902889 28321112 PMC5370389

[11] Chen Y Wu W. Determination of low level HBsAg in serum by microparticle enzyme immunoassay. Hepatobil Pancreat Dis Int. (2002) 1:262–4. Available online at: http://www.hbpdint.com/EN/Y2002/V1/I2/262 14612280

[12] Wang T Dai Y Zhang M Cui D Xu X Sun C. Sequence analysis of the Pre-S gene in chronic asymptomatic HBV carriers with low-level HBsAg. Int J Mol Med. (2018) 42:2689–99. 10.3892/ijmm.2018.3831 30132518 PMC6192773

[13] Xiang KH Michailidis E Ding H Peng YQ Su MZ Li Y. Effects of amino acid substitutions in hepatitis B virus surface protein on virion secretion, antigenicity, HBsAg and viral DNA. J Hepatol. (2017) 66:288–96. 10.1016/j.jhep.2016.09.005 27650283 PMC5523976

[14] Pollicino T Amaddeo G Restuccia A Raffa G Alibrandi A Cutroneo G. Impact of hepatitis B virus (HBV) preS/S genomic variability on HBV surface antigen and HBV DNA serum levels. Hepatology. (2012) 56:434–43. 10.1002/hep.25592 22271491

[15] Jia W Qi X Ji YY Xun YH Wang H Zhang WH. Low serum hepatitis B surface antigen level predicts compensated cirrhosis caused by chronic hepatitis B in HBeAg positive patients in east China. Hepat Mon. (2015) 15:e29183. 10.5812/hepatmon.29183 26322110 PMC4546813

[16] Villar LM Amado LA de Almeida AJ de Paula VS Lewis-Ximenez LL Lampe E. Low prevalence of hepatitis B and C virus markers among children and adolescents. Biomed Res Int. (2014) 2014:324638. 10.1155/2014/324638 25093164 PMC4100382

[17] Kim H Lee SA Won YS Lee H Kim BJ. Occult infection related hepatitis B surface antigen variants showing lowered secretion capacity. World J Gastroenterol. (2015) 21:1794–803. 10.3748/wjg.v21.i6.1794 25684944 PMC4323455

[18] Chook JB Teo WL Ngeow YF Tee KK Ng KP Mohamed R. Universal primers for detection and sequencing of hepatitis B virus genomes across genotypes A to G. J Clin Microbiol. (2015) 53:1831–5. 10.1128/JCM.03449-14 25788548 PMC4432068

[19] Jeffery-Smith A Hubb J Oliver A Tong CY. An apparent low level of hepatitis B surface antigen (HBsAg) in the presence of significant viral replication. J Clin Virol. (2016) 77:111–4. 10.1016/j.jcv.2015.11.030 26705961

[20] Yip TC Wong GL. Current knowledge of occult hepatitis B infection and clinical implications. Semin Liver Dis. (2019) 39:249–60. 10.1055/s-0039-1678728 30912100

[21] Liao H Liu Y Chen J Ding W Li X Xu Z. Characterization of hepatitis B virus (HBV) preS/S gene mutations in blood donors with occult HBV infection in the Baoji area of North China. Transfusion. (2017) 57:857–66. 10.1111/trf.14046 28236303

[22] Kuhns MC Holzmayer V Anderson M McNamara AL Sauleda S Mbanya D. Molecular and serological characterization of hepatitis B virus (HBV)-positive samples with very low or undetectable levels of HBV surface antigen. Viruses. (2021) 13:2053. 10.3390/v13102053 34696483 PMC8537069

[23] Phan NMH Faddy HM Flower RL Dimech WJ Spann KM Roulis EV. Low genetic diversity of hepatitis B virus surface gene amongst Australian blood donors. Viruses. (2021) 13:1275. 10.3390/v13071275 34208852 PMC8310342

[24] Adesina OA Akanbi OA Opaleye OO Japhet MO Wang B Oluyege AO. Detection of Q129H immune escape mutation in apparently healthy hepatitis B virus carriers in southwestern Nigeria. Viruses. (2021) 13:1273. 10.3390/v13071273 34210073 PMC8310067

[25] Yin Y Zhang P Tan Z Zhou J Wu L Hou H. The association of Pre-S/S gene mutations and hepatitis B virus vertical transmission. Hepat Mon. (2016) 16:e32160. 10.5812/hepatmon.32160 27226799 PMC4876664

[26] Shi Y Wei F Hu D Li Q Smith D Li N. Mutations in the major hydrophilic region (MHR) of hepatitis B virus genotype C in North China. J Med Virol. (2012) 84:1901–6. 10.1002/jmv.23419 23080494 PMC3747822

[27] Kumar M Sarin SK Hissar S Pande C Sakhuja P Sharma BC. Virologic and histologic features of chronic hepatitis B virus-infected asymptomatic patients with persistently normal ALT. Gastroenterology. (2008) 134:1376–84. 10.1053/j.gastro.2008.02.075 18471514

[28] Reijnders JG Rijckborst V Sonneveld MJ Scherbeijn SM Boucher CA Hansen BE. Kinetics of hepatitis B surface antigen differ between treatment with peginterferon and entecavir. J Hepatol. (2011) 54:449–54. 10.1016/j.jhep.2010.07.046 21112655

[29] Wu J Guo N Zhang X Xiong C Liu J Xu Y. HEV-LFS: a novel scoring model for patients with hepatitis E virus-related liver failure. J Viral Hepat. (2019) 26:1334–43. 10.1111/jvh.13174 31294523

[30] Pan L Zhang Y Xu Y Cao H Li L. Characteristics of CD^8+^ and CD^4+^ tissue-resident memory lymphocytes in the gastrointestinal tract. Adv Gut Microbiome Res. (2022) 2022:9157455. 10.1155/2022/9157455

[31] Dai Y Che F Jiang X Cui D Zhou H Xu X. Clinical characteristics and association analysis of persistent low-level HBsAg expression in a physical examination population with HBV infection. Exp Ther Med. (2020) 19:19–32. 10.3892/etm.2019.8217 31853269 PMC6909745

[32] Chen J Liu Y Zhao J Xu Z Chen R Si L. Characterization of novel hepatitis B virus PreS/S-gene mutations in a patient with occult hepatitis B virus infection. PLoS ONE. (2016) 11:e0155654. 10.1371/journal.pone.0155654 27182775 PMC4868315

[33] Nie JJ Sun KX Li J Wang J Jin H Wang L. A type-specific nested PCR assay established and applied for investigation of HBV genotype and subgenotype in Chinese patients with chronic HBV infection. Virol J. (2012) 9:121. 10.1186/1743-422X-9-121 22716091 PMC3477104

[34] Wu J Yu Y Dai Y Zhang Y Cheng J. Research progress on the mechanism of persistent low-level HBsAg expression in the serum of patients with chronic HBV infection. J Immunol Res. (2022) 2022:1372705. 10.1155/2022/1372705 35465353 PMC9020929

[35] Dai Y Hu Z Chen Y Lou B Cui D Xu A. A novel general and efficient technique for dissociating antigen in circulating immune complexes. Electrophoresis. (2018) 39:406–16. 10.1002/elps.201700246 28972666

[36] Kumar S Stecher G Li M Knyaz C Tamura K. MEGA X molecular evolutionary genetics analysis across computing platforms. Mol Biol Evol. (2018) 35:1547–9. 10.1093/molbev/msy096 29722887 PMC5967553

[37] Wu J Chen ZP Shang AQ Wang WW Chen ZN Tao YJ. Systemic bioinformatics analysis of recurrent aphthous stomatitis gene expression profiles. Oncotarget. (2017) 8:111064–072. 10.18632/oncotarget.22347 29340037 PMC5762305

[38] Xiang K Xiao Y Li Y He L Wang L Zhuang H. The effect of the hepatitis B virus surface protein truncated sC69* mutation on viral infectivity and the host innate immune response. Front Microbiol. (2019) 10:1341. 10.3389/fmicb.2019.01341 31249567 PMC6584109

[39] Ren F Yang X Hu ZW Wong VKW Xu HY Ren JH. Niacin analogue, 6-Aminonicotinamide, a novel inhibitor of hepatitis B virus replication and HBsAg production. EBioMedicine. (2019) 49:232–46. 10.1016/j.ebiom.2019.10.022 31680002 PMC6945246

[40] Mak LY Seto WK Fung J Yuen MF. Use of HBsAg quantification in the natural history and treatment of chronic hepatitis B. Hepatol Int. (2020) 14:35–46. 10.1007/s12072-019-09998-5 31745711

[41] Martinot-Peignoux M Asselah T Marcellin P. HBsAg quantification to optimize treatment monitoring in chronic hepatitis B patients. Liver Int. (2015) 5:82–90. 10.1111/liv.12735 25529092

[42] Tsai JF Margolis HS Jeng JE Ho MS Chang WY Hsieh MY. Immunoglobulin- and hepatitis B surface antigen-specific circulating immune complexes in chronic hepatitis B virus infection. Clin Immunol Immunopathol. (1998) 86:246–51. 9557157 10.1006/clin.1997.4477

[43] Oluyinka OO Tong HV Bui Tien S Fagbami AH Adekanle O Ojurongbe O. Occult hepatitis B virus infection in nigerian blood donors and hepatitis B virus transmission risks. PLoS ONE. (2015) 10:e0131912. 10.1371/journal.pone.0131912 26148052 PMC4492924

[44] Kim YJ Cho HC Choi MS Lee JH Koh KC Yoo BC. The change of the quantitative HBsAg level during the natural course of chronic hepatitis B. Liver Int. (2011) 31:817–23. 10.1111/j.1478-3231.2011.02516.x 21645212

[45] Bui TTT Tran TT Nghiem MN Rahman P Tran TTT Dinh MNH. Molecular characterization of hepatitis B virus in Vietnam. BMC Infect Dis. (2017) 17:601. 10.1186/s12879-017-2697-x 28859616 PMC5580302

